# Immunohistochemical Characterization of Langerhans Cells in the Skin of Three Amphibian Species

**DOI:** 10.3390/biology13040210

**Published:** 2024-03-23

**Authors:** Giorgia Pia Lombardo, Anthea Miller, Marialuisa Aragona, Emmanuele Messina, Angelo Fumia, Michał Kuciel, Alessio Alesci, Simona Pergolizzi, Eugenia Rita Lauriano

**Affiliations:** 1 Department of Chemical, Biological, Pharmaceutical, and Environmental Sciences, University of Messina, 98166 Messina, Italy; giorgiapia.lombardo@libero.it (G.P.L.); emmanuele.messina@outlook.com (E.M.); aalesci@unime.it (A.A.); elauriano@unime.it (E.R.L.); 2 Department of Veterinary Sciences, University of Messina, Polo Universitario dell’Annunziata, 98168 Messina, Italy; mlaragona@unime.it; 3 Department of Clinical and Experimental Medicine, University of Messina, 98124 Messina, Italy; angelofumia@gmail.com; 4 Poison Information Centre, Department of Toxicology and Environmental Disease, Faculty of Medicine, Jagellonian University, Kopernika 15, 30-501 Krakòw, Poland; michalkuciel@gmail.com

**Keywords:** amphibia, Anura, Caudata, Apoda, skin Langerhans cells

## Abstract

**Simple Summary:**

Amphibians are classified into three orders with different morphological characteristics: Anura, Apoda, and Caudata. In this article, the importance of skin for amphibians is discussed as it represents a physical, chemical, immunological, and microbiological barrier to pathogen insult is discussed. Langerhans cells (LCs) are antigen-presenting cells constituting an important component of the immune system in the skin. This study aims to characterize Langerhans cells in the skin of *Lithobates catesbeianus* (Shaw, 1802), *Amphiuma means* (Garden, 1821), and *Typhlonectes natans* (Fischer, 1880) with several antibodies previously used to mark the same cells in other vertebrates. The results showed that the distribution of LCs is very similar in the three amphibian species examined, despite their different habitats. In conclusion, the amphibian dendritic cells are morphologically and immunohistochemically homologous to the LCs of all vertebrates so they can be considered as starting points to better understand the phylogeny of the vertebrate immune system.

**Abstract:**

The amphibian taxon includes three orders that present different morphological characteristics: Anura, Caudata, and Apoda. Their skin has a crucial role: it acts as an immune organ constituting a physical, chemical, immunological, and microbiological barrier to pathogen insult and conducts essential physiological processes. Amphibians have developed specialized features to protect the vulnerable skin barrier, including a glandular network beneath the skin surface that can produce antimicrobial and toxic substances, thus contributing to the defense against pathogens and predators. This study aims to characterize Langerhans cells in the skin of *Lithobates catesbeianus* (order: Anura; Shaw, 1802), *Amphiuma means* (order: Caudata; Garden, 1821), and *Typhlonectes natans* (order: Apoda; Fischer, 1880) with the following antibodies: Langerin/CD207 (c-type lectin), Major Histocompatibility Complex (MHC)II, and Toll-like receptor (TLR)2 (expressed by different types of DCs). Our results showed Langerhans cells positive for Langerin CD/207 in the epidermis of the three species; moreover, some antigen-presenting cells (APCs) in the connective tissue expressed TLR2 and MHCII. The distribution of the Langerhans cells is very similar in the three amphibians examined, despite their different habitats. A greater knowledge of the amphibian immune system could be useful to better understand the phylogeny of vertebrates and to safeguard amphibians from population declines. Furthermore, the similarities between amphibians’ and human skin concerning immunological features may be useful in both biology and translational medicine.

## 1. Introduction

The amphibian taxon represents a link between fish and other tetrapods and includes three orders: Anura or Salientia (frogs and toads), Caudata or Urodela (salamanders), and Apoda or Gymnophiona (caecilians). In this study, three species, each belonging to one of three orders of amphibians, have been considered: *Lithobates catesbeianus* (Shaw, 1802), *Typhlonectes natans* (Fischer, 1880), and *Amphiuma means* (Garden, 1821).

*L. catesbeianus* (also known as *Rana catesbeiana* or American bullfrog) belongs to the Anura order; it is native to North America and normally inhabits swamps and lakes, but it can also be found in manmade habitats such as canals and ditches. *T. natans* is a species belonging to the Gymnophiona order and Caeciliidae family. It is endemic to northwestern South America and its habitat includes mostly dry savannas, rivers, and lakes. *A. means* is a caudate amphibian endemic to the southeastern USA; it usually lives in ditches, swamps, streams, and ponds. Amphibian skin, because it relies on terrestrial or aquatic habitats or a combination of both, is an extremely varied and adaptable organ [1] which performs several physiological functions such as sensory perception, camouflage, ionic transport, and respiration [2]; furthermore, it represents a mechanical and chemical barrier against pathogens [3,4,5].

Amphibian skin consists of three consecutive layers: epidermis, dermis, and hypodermis. The epidermis is organized into four cell layers; from the innermost to the outermost part, there is the germinative layer (*stratum germinativum*), consisting mainly of epithelial cells, immune cells [6], and chromatophores [7]; the spinous layer (*stratum spinosum*), composed of terminally differentiating cells; the granulous layer (*stratum granulosum*); and the hard layer (*stratum corneum*), composed of a very thin layer of keratinized cells [8,9].

The epidermal layers include the basal lamina (basement membrane), which separates the epidermis from the dermis [10]. The amphibian dermis is commonly composed of a vascularized *stratum spongiosum* of loose connective tissue containing chromatophores, blood vessels, alveolar mucous and granular glands, and a *stratum compactum* formed by collagenous fibers organized in intersecting lines [11,12]. The hypodermis (the subcutaneous layer) is a layer below the dermis composed of loose connective tissue. Amphibian skin can perform the above-mentioned functions due to the presence of the glands, localized in the spongious dermal layer [13]. The most ubiquitous glands in amphibian skin are mucosal, granular, and mixed glands, which combine mucous and granular secretion [14,15]. Mucosal glands produce mucus, which performs various functions such as protecting the amphibian against mechanical damage and bacterial or fungal agents, keeping the skin moist, and slowing down water loss [16,17]. Granular glands contain bioactive molecules, such as antimicrobial peptides (AMPs) and toxic alkaloids, involved in host and predator defense [15,18,19]. Amphibian skin is also an immunological barrier because it contains several immune cells, such as mast cells (MCs) [20,21], and Langerhans cells (LCs) [22]; these represent the main cellular components of the skin immune system, while few studies report the presence of macrophages and lymphocytes in healthy amphibian skin tissue [23]. MCs are effector cells of allergic and other inflammatory reactions; their involvement in innate and adaptive immunity particularly concerns leukocyte recruitment and stimulation of T-cell migration [24]. LCs are immature dendritic cells able to identify foreign antigens such as pathogens and bacteria [25,26]. They are localized in the epidermis and are considered the most peripheral component of the immune system; their function is linked to the uptake and presentation of antigens to T lymphocytes like antigen-presenting cells (APCs), to initiate a cellular or humoral immune response [27,28]. LCs are present in all vertebrates; these immune cells express pattern recognition receptors (PRRs) whose link with pathogen-associated molecular patterns (PAMPs) leads to an innate amplifying inflammatory cascade [29]. In a previous study, LCs were characterized through enzymatic histochemistry for ATPase in *Rana catesbeiana* [30]. Furthermore, co-expression of MHC class II molecules in ATPase-positive epidermis dendritic cells was demonstrated also in *Rana catesbeiana* [31] and *Lithobates pipiens* (Schreber, 1782) [32]. No studies have been reported in the literature on the cells of the peripheral immune system in Caudata and Apoda skin [31].

Langerin/CD207 is a c-type lectin [33,34] detectable in different cell types such as LCs and dendritic cells [35,36] in most epithelial and connective tissues. In previous studies, Langerhans-like dendritic cells have been characterized using the Langerin/CD207 antibody in the lung and airways and associated lymph nodes of *Stenella coeruleoalba* (Meyen, 1833) [37]. Moreover, Langerin/CD207 has been used to characterize dendritic cells in the spiral valve and intestine of the elasmobranch *Scyliorhinus canicula* (Linnaeus, 1758) [38,39] and the intestinal dendritic cells of several fish species: *Eptatretus cirrhatus* (Forster, 1801), *Polypterus senegalus* (Cuvier, 1829), *Lepisosteus oculatus* (Winchell, 1864), and *Clarias batrachus* (Linnaeus, 1758) [39].

Toll-like receptors (TLRs) are one of the most highly conserved PRRs in the animal kingdom; TLR2 is particularly relevant for vertebrate immunity because it is the only TLR capable of forming functional heterodimers with more than two other types of TLRs [40,41]. TLR2 has been reported in the macrophages and dendritic cells of amphibian fish such as *Periophthalmodon schlosseri* (Pallas, 1770) [42]. Some TLR genes have been identified in a few frog species; the TLR2 family is present in *Xenopus tropicalis* (Gray, 1864) [23].

Major Histocompatibility Complex class II (MHCII) molecules are critical in the control of many immune responses, helping dendritic cells, macrophages, and a few limited other cell types after appropriate stimulation in the antigen recognition [43]. This study aims to characterize for the first time Langerhans cells in the skin of the three amphibian species through immunohistochemistry techniques, using the same antibodies previously used to detect the same cells in other vertebrates, thus demonstrating a phylogenetic continuity in the development of the vertebrate immune system. A more comprehensive study about the integrity of the amphibian skin related to the health of these animals could be relevant to understand the immune cell composition of amphibian cutaneous tissues that still remains unexplored. In addition, considering the recent amphibian population declines, it might be interesting to understand the natural and potential manmade environmental changes that could modulate amphibian immune function.

## 2. Materials and Methods

### 2.1. Experimental Protocol

Samples of the integument of *Lithobates catesbeianus, Amphiuma means,* and *Typhlonectes natans* come from our laboratory histotheca and were prepared using the standard methods to arrange long-lasting preparations for light microscopy.

### 2.2. Histology and Immunohistochemistry

Integumental samples from the dorsal and lateral body walls were cut into 5 μm sections. Hematoxylin and eosin (H&E), Mallory trichrome, and Alcian blue-periodic acid Schiff (AB-PAS; pH 2.5) staining were used on the sections, which were analyzed using a Zeiss Axioskop 2 plus microscope equipped with an Alexasoft TP3100A CMOS Digital camera (Alexasoft, Florence, Italy).

### 2.3. Immunoperoxidase Method

Immunohistochemical studies were carried out using the peroxidase indirect technique. Serial sections were incubated in a phosphate-buffered saline solution for 30 min to prevent the activity of endogenous peroxidase; then, to rinsed sections, normal goat antiserum (1:20; Sigma, St. Louis, MO, USA) was added. Serial sections were incubated overnight at 4 °C in a humid chamber with mouse antibody to Langerin/CD207 (Santa Cruz Biotechnology, Inc. Dallas, TX, USA).

After washing in PBS, the sections were incubated for 2 h with a goat anti-mouse IgG-peroxidase conjugate (Sigma-Aldrich, St. Louis, MO, USA). The slices were incubated for 1–5 min at room temperature in a solution of 0.02% diaminobenzidine (DAB) and 0.015% hydrogen peroxide to detect peroxidase activity. Sections were dehydrated, mounted, and studied using an Axiophot Zeiss microscope equipped with an Alexasoft TP3100A CMOS Digital camera, after being rinsed in PBS. Control experiments were performed excluding primary antibodies.

### 2.4. Confocal Immunofluorescence

Serial slices previously deparaffinized and rehydrated (5 μm thick) were rinsed with 0.2% Triton-X 100 in PBS solution (pH 7.5). After incubating the slices in a 0.3% H_2_O_2_ solution to inhibit endogenous peroxidase activity, the washed sections were treated for 30 min with bovine serum albumin (F7524, Sigma-Aldrich, St. Louis, MO, USA) to inhibit nonspecific binding, and then the primary antibodies were incubated. Anti-Langerin/CD207 and anti-MHC class II (Y-Ae) monoclonal antibodies were used in the double-label experiments, with a polyclonal antibody for TLR2 (for details, see Table 1). A humid chamber was used for overnight incubation at 4 °C. The sections were then washed in buffer and incubated for 40 min at room temperature with Alexa Fluor IgG (H + L) secondary antibodies (for details, see Table 1) in a dark, humid chamber. Lastly, the dehydrated sections were mounted using Fluoromount Aqueous Mounting Medium (Sigma-Aldrich, Burlington, MA, USA).

A Zeiss LSMDUO confocal laser scanning microscope with META module (Carl Zeiss Micro Imaging GmbH, Oberkochen, Germany) was used to analyze the sections and acquire images. Every image was taken quickly in order to reduce photodegradation. Digital images were cropped, and the figure montage was prepared using Adobe Photoshop 2023 (Adobe Systems, San Jose, CA, USA).

### 2.5. Quantitative Analysis

To collect data for the quantitative analysis, a total of 6000 fields (10 samples of skin per specimen, 10 sections per sample, 20 fields per section) were examined. ImageJ software 1.53e was used to evaluate the quantity and positivity of cells. SigmaPlot version 14.0 (Systat Software, San Jose, CA, USA) was used to count the number of LCs positive for Langerin/CD207, MHCII, and TLR2. One-way ANOVA was used to assess the normally distributed data (verified by Shapiro–Wilk test; *p* < 0.001). The number of immuno-reactive LCs is expressed as the mean values and standard deviations (SD).

## 3. Results

### 3.1. Histological and Histochemical Description of the Skin

**Figure 1:**
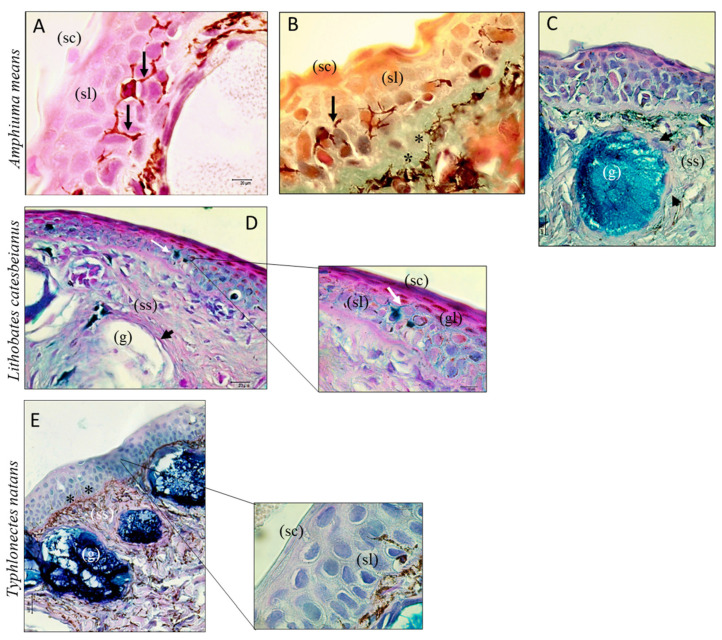
Sections of *Amphiuma means* (**A**–**C**), *Lithobates catesbeianus* (**D**), and *Typhlonectes natans* (**E**) skin; 40×, scale bar 20 µm. In the epidermis, the *stratum corneum* (sc), granulous layer (gl), and spinous layer (sl) are clear (**A**,**B** and in inserts of **D**,**E**). Large-sized glands (g) are observed in the stratum spongiosum (ss) of the three Amphibian species with AB/PAS staining; some myoepithelial cells surrounding glands acini have been detected (arrowhead). Among the skin layers, the stratum compactum can be clearly distinguished; indeed, its collagen fibers are marked in blue by Mallory staining (**B**). Furthermore, in this image, numerous melanophores are visible in the dermis (*). LCs stained with H/E and Mallory are characterized by ramifications surrounding the keratinocytes (black arrows) (**A**,**B**). LCs have also been marked with AB/PAS staining (**D**) (white arrow); Insert: magnification of an LC present in Figure (**D**).

The skin of the three species considered consists of epidermal and dermal layers. Different epidermis cells were stained with H&E, Mallory, and AB/PAS (Figure 1). The outermost layer of the epidermis is thinner and it is composed of keratinized cells (*stratum corneum*), which mainly have a protective function. Under this layer, cells with a tendentially cubic shape can be observed; they constitute the spinous layer (Figure 1A,D,E). Several glands were identified in the *stratum spongiosum* (dermis) with AB/PAS staining; the intensity of the AB reaction is particularly strong in the adenomere because of the presence of acid glycosaminoglycans (Figure 1C,D,E). The acini of these glands appeared to be surrounded by myoepithelial cells (Figure 1C), already previously marked with α-SMA in *Typhlonectes natans* skin during a study conducted by Zaccone (2014) [44]. The histological microscope conducted in this study showed morphological features not described previously in amphibians; indeed, between keratinocytes, there are numerous dendritic-like cells with a characteristic morphology (branched structure) resembling LCs, as revealed by Carrillo Farga (1990) [30] using enzymatic techniques. In Langerhans cells stained with H/E and Mallory, indeed it is possible to observe their ramifications which surround the keratinocytes and sometimes run parallel to the membranes of the same cells (Figure 1A,B). AB/PAS staining marks c-type lectin in blue, so considering that Langerin is a c-type lectin which is expressed by Langerhans cells, it is further confirmed that the cells marked in Figure 1D are LCs. The amphibian dermis is commonly composed of a vascularized stratum spongiosum of loose connective tissue containing chromatophores (Figure 1B,E).

### 3.2. Immunohistochemistry and Confocal Scanning Laser Microscopy

**Figure 2:**
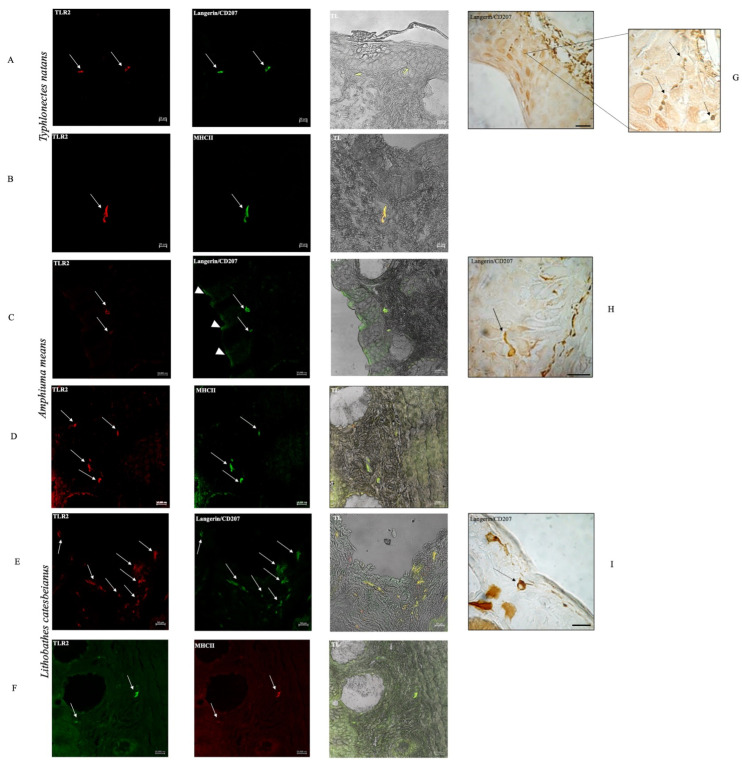
Immunoperoxidase and immunofluorescence of TLR2, Langerin/CD207, and MHCII in *Typhlonectes natans, Amphiuma means,* and *Lithobates catesbeianus* skin.; 40×, scale bar 20 µm. Immunohistochemical characterization of LCs that are Langerin CD/207 positive in *Typhlonectes natans, Amphiuma means,* and *Lithobates catesbeianus* skin (**G**–**I**) (black arrows). Confocal immunopositivity of LCs for TLR2 (red) and Langerin/CD207 (green) in the loose connective tissue of *Typhlonectes natans* and *Lithobates catesbeianus* is evident (**A**,**C**,**E**) (white arrows). In *Amphiuma means,* some LCs have been revealed to be positive for TLR2 (red) and Langerin/CD207 (green) both in the superficial connective tissue (white arrows) and in the epidermis (arrowheads) (**C**). In addition, some LCs positive for TLR2 and MHCII have been observed in the deep connective tissue of the three species (**B**,**D**,**F**) (arrows). It is interesting to note that the change in the form of LCs corresponds to their function (see discussions). Moreover, some LCs that are Langerin/CD207 positive have been detected in the epidermis of all three species through an immunohistochemical peroxidase reaction; 40×, scale bar 20/50 μm. TL =  transmitted light.

The results observed through a confocal microscope were found to be similar among the three species considered. Some LCs that were Langerin/CD207 positive were marked in the epidermis of *Typhlonectes natans*, *Amphiuma means*, and *Lithobathes catesbeianus* in immunohistochemical peroxidase investigations (Figure 2G–I). The immunofluorescence with anti TLR2 and Langerin/CD207 revealed some pseudo-circular/oval-shaped cells in the superficial connective tissue, strongly reactive in expression and colocalization, in *Typhlonectes natans, Lithobathes catesbeianus*, and *Amphiuma means* (Figure 2A,C,E). In the latter, some LCs positive for TLR2 and Langerin/CD207 were also detected in the epidermis. In the deep connective tissue of the three species, some elongated cells were positive for both TLR2 and MHCII (Figure 2B,D,F). All antibodies used characterized Langerhans cells that can change shape concerning the function they perform. The TLR2 confocal results countered those obtained with the immunohistochemical peroxidase investigations.

### 3.3. Quantitative Analysis

Quantitative analysis revealed a similar number of LCs in all the skin samples of the amphibian species examined (Table 2). No significative difference in the number of LCs between the three clusters has been determined.

## 4. Discussion

Among existing animals, amphibians are the first tetrapods that adapted to terrestrial life from aquatic environments [14]. They have adjusted to many different habitats in terms of morphological and physiological traits; as animals with bare, permeable skin, they need to be kept moderately moist for skin respiration and to maintain their body temperature [8,45]. Amphibians share some characteristics with other vertebrates, including mammals, such as skin structure and its immune system components. Skin-associated lymphoid tissue (SALT), for instance, is a specific immunologic environment shared by all vertebrates composed of immune cells and molecules [46].

Important molecular components of the amphibian innate immunity are AMPs which constitute a first barrier against a wide variety of pathogens [22,47,48]. Amphibian skin AMPs have a significant activity against antibiotic-resistant bacteria, protozoa, and fungi by permeating and destroying the plasma membrane and inactivating intracellular targets [47,49]. AMPs do not bind to a specific receptor, so they are less likely to activate resistance mechanisms [50,51]. Moreover, some peptides with antitumoral activity but little toxicity against non-malignant cells have been found in amphibian skins [52].

In amphibian skin, lymphocytes and accessory cells such as macrophages, mast cells (MCs), dendritic cells, and Langerhans cells (LCs) have been described [53,54,55,56]. MCs in frog tissues have been reported as displaying a close association with blood vessels, nerve fiber bundles, and melanocytes [20]. They play a key role in inflammatory responses through the degranulation of biologically active compounds such as histamine, both in amphibians and in other vertebrates [57,58,59] LCs and MCs are located in amphibian skin even under normal conditions, whereas reports on the presence of macrophages and lymphocytes in healthy skin tissue are rare [60]. LCs are defined as peripheral immune surveillance cells because they are mainly positioned at the environmental barrier; they are ubiquitous in the epidermis, where they form a dense three-dimensional network. Moreover, they can constantly explore the environment by extending and retracting their dendrites to spot any pathogen entering the skin barrier [61,62]. LCs in amphibian skin have been previously studied by Mescher in 2007 [63], who detected these cells in the epidermis and wound epithelia of regenerating limbs in both urodeles and *Xenopus* larvae. In addition, LCs were detected based on the presence of epidermal cells positive for ATPase activity, vimentin, and major histocompatibility complex class II antigens in *Xenopus laevis* (Daudin, 1802) [63,64]. This research demonstrates that it is possible to characterize LCs in the three orders of amphibians with the same antibodies as previously used to mark these cells in other vertebrates.

Comparative immunology usually focuses on the major differences in the immune system between different organisms in order to draw conclusions on the evolution of immunity, but it is also important to highlight the similarities. Detecting Langerhans cells in amphibian skin not only gives us additional information on their immune system components but also represents a bridge between amphibians and other vertebrates, allowing the merging of several research lines. Increasing knowledge about amphibians and their immune systems, indeed, can be useful for several reasons, including to deepen some fundamental notions about primary physiological processes such as organ development and tissue regeneration, cell growth, neuron functioning, metabolism, and differentiation (these processes involve various components of the immune system) [65,66,67,68].

Our findings show that the distribution of LCs is very similar in the three amphibian species examined, despite their different aquatic and terrestrial habitats. The results of this study show a very interesting feature of LCs: they change shape and consequently function during the performance of their immune function. LCs acquire an elongated shape in the deep connective tissue, while they have a pseudo-circular shape in the superficial connective tissue and the epidermis. Furthermore, while in the epidermis they are positive for TLR2 and Langerin/CD207 (the last is a selective marker of immature LCs) [69,70,71], in the connective tissue they are positive for both TLR2 and MHCII (which specifically marks cells exposing the processed antigen) [72]. It is interesting to observe that LCs maintain their positivity for TLR2; this happens because it marks immune cells in general.

## 5. Conclusions

In conclusion, the amphibian dendritic cells characterized in this study are morphologically and immunohistochemically homologous to the LCs of all vertebrates. Given the essential role of LCs in bridging innate and acquired immune systems, these cells can be considered as starting points to better understand the phylogeny of the vertebrate immune system. Furthermore, the study of amphibians offers many fascinating insights into several areas of research concerning skin [8] and kidney [73] diseases which may be useful in the biology and medicine fields. Moreover, considering the recent emergence of resistance to commercially available antibiotics/antimycotics, the discovery of alternative anti-infective agents is necessary, and amphibian skin AMP derivatives represent valid candidates for the development of new antimicrobial compounds with expanded properties, for both human and veterinary medicine [74,75,76]. Finally, deepening knowledge about the immune system of amphibians could be useful to develop strategies to assist disease mitigation and to safeguard amphibians from population declines, caused especially by infectious diseases. Carey et al. (1999) [77] indeed suggested that research concerning the immune defenses against a specific pathogen should include studies of both innate and adaptive immune defense mechanisms and should identify susceptible life stages. It could also be relevant to understand the mechanism of spreading pathogens, how they penetrate the skin, and whether these microorganisms can infect and replicate faster than the immune system can set up an effective defense. If future studies focused on how other natural and anthropogenic conditions influence the interaction between pathogens and the amphibian immune system, it would be easier to find solutions in order to prevent the decline of amphibian populations.

## Figures and Tables

**Table 1:** Antibody data.

Primary Antibodies	Supplier	Catalog Number	Source	Dilution
MHC class II (Y-Ae)	Santa Cruz biotechnology	sc-32247	mouse	1:300
TLR-2 (p-Ab)	Active Motif	40981	rabbit	1:200
Langerin/CD207	Santa Cruz biotechnology	sc-271272	mouse	1:300
**Secondary Antibodies**	**Supplier**	**Catalog Number**	**Source**	**Dilution**
Alexa Fluor 488 donkey anti-mouse IgG (H + L)	Invitrogen	A21202	donkey	1:300
Alexa Fluor 594 donkey anti-rabbit IgG (H + L)	Invitrogen	A21207	donkey	1:300

**Table 2:** Quantitative analysis results (*n* = 3) ^1^.

	*L. catesbeianus*	*A. means*	*T. natans*
	Mean ± SD	Mean ± SD	Mean ± SD
TLR2+	374.56 ± 23.78	363.47 ± 21.67	342.63 ± 25.74
Langerin/CD207+	323.25 ± 24.53	311.52 ± 23.41	312.63 ± 28.91
MHCII+	366.05 ± 24.73	356.28 ± 29.23	326.55 ± 22.32
TLR2 + Langerin/CD207+	314.29 ± 23.40	305.27 ± 22.45	308.20 ± 28.86
TLR2 + MHCII+	321.34 ± 21.04	317.49 ± 23.78	309.71 ± 31.92

^1^ One-way ANOVA was used to verify if there were differences between the three amphibians. *p* values for the One-way ANOVA ranged from 0.236 to 0.759. Shapiro–Wilk test was used to assess data normality and homogeneity. For the Shapiro–Wilk test, W values ranged from 0.789 to 0.991. *p* values were all <0.001.

## Data Availability

Data are contained within the article.
